# Comparative Analysis of Temperature Variations Following Sympathetic Blocks in Warm and Cold Subtypes of Complex Regional Pain Syndrome (CRPS): A Retrospective Cohort Study

**DOI:** 10.3390/jcm14062060

**Published:** 2025-03-18

**Authors:** Burcu Candan, Semih Gungor

**Affiliations:** 1 Division of Musculoskeletal and Interventional Pain Management, Department of Anesthesiology, Critical Care and Pain Management, Hospital for Special Surgery, New York, NY 10021, USA; 2 Department of Anesthesiology, Weill Cornell Medicine, New York, NY 10065, USA

**Keywords:** complex regional pain syndrome, warm CRPS, cold CRPS, lumbar sympathetic block, forward-looking infrared thermography, FLIR camera, thermal images

## Abstract

**Background/Objectives**: The pathophysiological mechanisms of temperature asymmetry differ between patients with warm and cold subtypes of Complex Regional Pain Syndrome (CRPS). Consequently, the response to lumbar sympathetic blocks (LSBs) and the resulting temperature improvement may vary between these two subtypes. We aimed to evaluate whether there was a significant difference in temperature elevation following sympathetic blocks in warm versus cold subtypes of CRPS. **Methods**: We calculated the temperature difference by analyzing forward-looking infrared (FLIR) thermal camera images of the affected extremity at pre-block and 5-min post-block time points. The primary outcome measure was that the mean temperature increase following LSB would be higher in the cold CRPS group than in the warm CRPS group. The secondary outcome measure was that the mean temperature elevation following the sympathetic block in the cold CRPS subtype would be at least 50% higher than in the warm CRPS subtype. **Results**: The study assessed warm and cold CRPS subtypes by analyzing temperature profiles from 90 lumbar sympathetic blocks performed on 34 patients. The temperature change in the affected extremity following LSB varied widely, with the highest increase observed in one patient at 10.99 °C. The cold CRPS patients demonstrated a higher mean temperature increase at the 5 min time point following LSB, averaging 3.37 °C in initial cases and 2.67 °C across all cases. In comparison, warm CRPS patients had lower mean increases of 0.58 °C in initial cases and 1.23 °C across all cases. Notably, the mean temperature rise in the cold CRPS group exceeded that of the warm CRPS group by more than 50%, meeting the secondary outcome goal. **Conclusions**: Our results indicated that patients with the cold subtype of CRPS tend to experience greater temperature improvements compared to those with the warm subtype after undergoing a sympathetic block. Therefore, our findings suggest that the criteria for determining the success of a sympathetic block should be revised to account for the cold and warm subtypes of CRPS.

## 1. Introduction

Skin temperature asymmetry between the affected and unaffected limbs is a common feature of Complex Regional Pain Syndrome (CRPS). CRPS is divided into two subtypes—warm and cold—based on the temperature of the affected limb at the time of diagnosis [1,2]. In the early stages of CRPS, the affected limb is typically warmer than the healthy limb due to inflammation associated with warm CRPS. However, in later stages, the affected limb may become colder [3]. In most patients receiving active therapy, the inflammatory component of warm CRPS usually resolves within approximately 12 months [4,5].

A majority of CRPS patients (about 75–80%) recover within 6–13 months, while around 20% may progress to the chronic cold stage [5,6,7]. Patients initially diagnosed with warm CRPS are more likely to recover than those diagnosed with cold CRPS [2]. Furthermore, the presence of cold CRPS at the initial presentation indicates a worse prognosis [8].

The “warm” and “cold” forms are informal classifications reflecting vasomotor dysfunction as the primary clinical feature. Approximately 70% of cases present as warm CRPS, while 30% present as cold CRPS [8].

There are several pathophysiological explanations for the temperature difference observed between limbs in patients with CRPS. Vascular abnormalities are prominent clinical features of CRPS that mainly impact microcirculation in the affected limb, leading to abnormal vasodilation, temperature asymmetry, and skin discoloration. In addition, inflammatory mechanisms contribute to the sensitization of nociceptors and enhance the adrenergic response, which can result in the vasoconstriction of peripheral blood vessels [2,3,4,5,6,7,8,9,10,11,12]. During the early phase of CRPS, vasodilation is predominantly observed in the affected limb, while vasoconstriction is more common in the chronic phase [11].

The warm subtype of CRPS is characterized by warm skin and swelling. The underlying mechanisms are related to an increased inflammatory response [4,13], the loss of vasoconstrictor reflexes originating from the central nervous system, and subsequent vasodilation that affects microcirculation [9]. This effect can be further intensified by neurogenic inflammation in the affected limb due to peripheral mechanisms [10]. Studies have shown that, on average, the affected side is 1.3 °C warmer than the unaffected side in the warm CRPS subtype [14]. In contrast, the cold CRPS subtype typically occurs in the chronic and later stages of the condition. It is associated with increased sympathetic activity, vasoconstriction, and reduced blood flow, presenting with cold and pale limbs [8,15].

The skin temperature asymmetry observed in CRPS is a dynamic phenomenon that fluctuates in response to changes in sympathetic activity [12]. Sympathetic blocks are frequently performed on CRPS patients for diagnostic or therapeutic purposes related to potentially sympathetically mediated pain [16]. The literature indicates that physiological inhibition of cutaneous sympathetic vasoconstrictor activity can lead to an increase in skin temperature. Additionally, applying a sympathetic blockade to the affected extremity can modulate overall sympathetic outflow [17]. As a result, following sympathetic blocks, an increase in circulation and a moderate rise in temperature in the affected extremity are expected. Since warm and cold CRPS patients have different mechanisms that contribute to their temperature variations, we hypothesize that their responses to lumbar sympathetic block and subsequent temperature improvements may also differ.

After a sympathetic block, we expect the temperature increase to be higher in patients with the cold subtype of CRPS due to vasoconstriction compared to those with the warm subtype. Therefore, we hypothesize that patients with cold CRPS experience a significantly greater elevation in temperature following sympathetic blocks than those with warm CRPS.

Real-time skin temperature measurement with forward-looking infrared (FLIR) imaging is a useful technique for evaluating the effectiveness of lumbar sympathetic blocks [18,19]. This study aimed to investigate the temperature change patterns between the warm and cold subtypes of CRPS following sympathetic blockade. Additionally, we assessed whether the temperature increase was significantly greater in the cold CRPS subtype after the sympathetic block.

## 2. Materials and Methods

This retrospective cohort study was conducted at a specialized urban academic center that specializes in treating musculoskeletal disorders and chronic pain. The study received approval from the Institutional Review Board (IRB approval number: 2023-1555). This study includes patients who meet the Budapest criteria for diagnosis of CRPS of the lower extremity within the study date and who presented for lumbar sympathetic blocks. We retrospectively analyzed the preoperative FLIR images obtained by the thermal camera in 90 consecutive lumbar sympathetic blocks performed on 34 CRPS patients from 1 February to 31 July 2023. We analyzed the results in two groups, including the “initial group” for 34 initial procedures and “all cases” for all 90 consecutive procedures.

### 2.1. Hypothesis

The temperature changes after sympathetic blocks are expected to differ between the warm and cold subtypes of CRPS due to the distinct pathophysiological mechanisms involved. Specifically, the temperature increase following a sympathetic block is anticipated to be significantly higher in the cold subtype due to pre-existing vasoconstriction compared to the warm subtype.

### 2.2. Primary and Secondary Outcome

#### 2.2.1. Primary Outcome

The primary outcome is to compare the temperature increase after lumbar sympathetic block between the cold and warm CRPS groups, with the expectation that the increase will be greater in the cold CRPS group.

#### 2.2.2. Secondary Outcome

The secondary outcome is to assess whether the mean temperature increase following the sympathetic block is at least 50% greater in the cold CRPS subtype compared to the warm CRPS subtype.

### 2.3. Patient Inclusion and Exclusion Criteria

#### 2.3.1. Patient Inclusion Criteria

The patient selection criteria of this study are as follows:

- The patient is between 18 and 85 years old.
- They meet CRPS diagnostic criteria by using the Budapest Clinical Diagnostic Criteria.
- The patient has had pain and other symptoms and signs for more than 3 months.
- The patient is not responding to conventional medical treatments and a multidisciplinary approach that includes analgesic support (NSAIDs, opioids, and topical analgesics), tricyclic antidepressants (TCAs), serotonin-norepinephrine reuptake inhibitors (SNRIs) such as duloxetine, anticonvulsants (gabapentin, pregabalin), physical therapy, psychological support, and other adjunctive therapies.
- The patient has a high NRS (Numerical Rating Scale) detection in pain assessment despite appropriate treatment (NRS ≥ 6/10).

#### 2.3.2. Patient Exclusion Criteria

The patient exclusion criteria of this study are based on the following criteria:

- Patients with suspected disk herniation, spinal stenosis, myelopathy, and suspected radiculopathy in detailed examinations and examinations [MRI (magnetic resonance imaging), CT (computerized tomography)].
- Systemic or local infection.
- Coagulation disorders.
- History of allergy to contrast material.
- Malignancy.
- Pregnancy.
- Uncontrollable medical and psychiatric condition.
- The patients diagnosed with dysautonomia, sympathetic dysfunction (such as Raynaud disease or Buerger disease), sweating disorders (such as acquired idiopathic generalized anhidrosis), and patients on vasoactive drugs, the mechanism of action is directly on the vascular tone.
- The patients require IV sedation/anesthesia.

Typically, lumbar sympathetic block interventions for CRPS patients were scheduled for three consecutive sympathetic blocks, spaced 1–3 weeks apart, based on observed benefits in our practice. Treatment was discontinued prematurely if the patient did not appear to benefit from LSB. All consecutive lumbar sympathetic blocks for each patient were included in the current study.

### 2.4. Pre-Procedural Approach

All procedures were performed under local anesthesia. Baseline FLIR thermal camera images were captured before the LSB after acclimating to a climate-controlled room at 21 °C.

**Figure 1:**
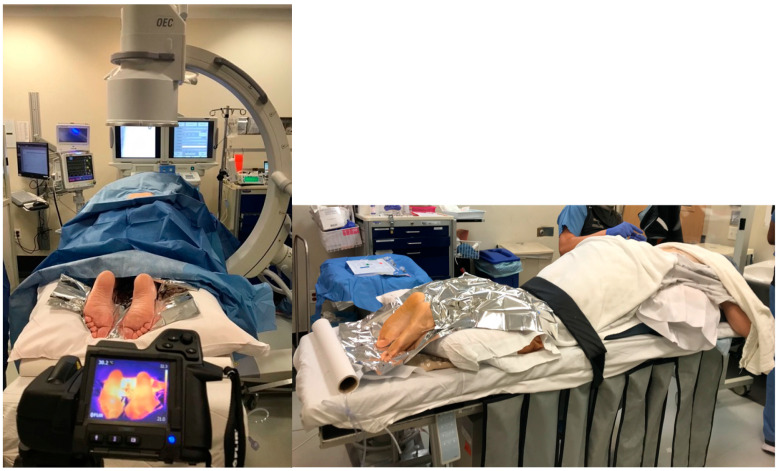
The position of the patient and the thermal camera.

Before the procedure, we captured a thermal image of the affected lower extremity via a standardized process. Each image was taken at a perpendicular angle, and the feet were separated from the background with the help of a Mylar blanket placed behind the feet (Figure 1). This process helped the software effectively isolate the feet from the background in the thermal image, which was critical in accurately measuring the temperature profile of the feet.

All CRPS patients enrolled in this study have undergone planned LSB in the prone position following necessary monitoring in the operating room (Figure 1). C-arm fluoroscopy imaging was utilized during the procedure to ensure precise needle localization for effective sympathetic blockade. After sterilizing the patient’s lower back, appropriate covering was applied to maintain a sterile environment throughout the entire procedure. The fluoroscopy provided aid in determining the appropriate procedural level. Throughout the procedure, fluoroscopy imaging guided the advancement of a 22-gauge, 5- or 7-inch needle to the anterolateral surface of the L3 vertebral body. Upon reaching the targeted position in the imaging, confirmation of the absence of body fluid was evaluated through negative needle aspiration. At this stage, to validate needle localization, 4 mL of contrast material was administered, and the appropriate spread of this contrast agent on the anterolateral surface of the L3 vertebral body was observed. Subsequently, 10 mL of Bupivacaine 0.5% was administered, with ongoing intermittent control through negative aspirations.

### 2.5. Post-Procedural Approach

After the procedure, we obtained a second FLIR image of the affected lower extremity at the 5th minute using the same technique via a standardized process as mentioned above.

### 2.6. FLIR Camera Measurements

**Figure 2:**
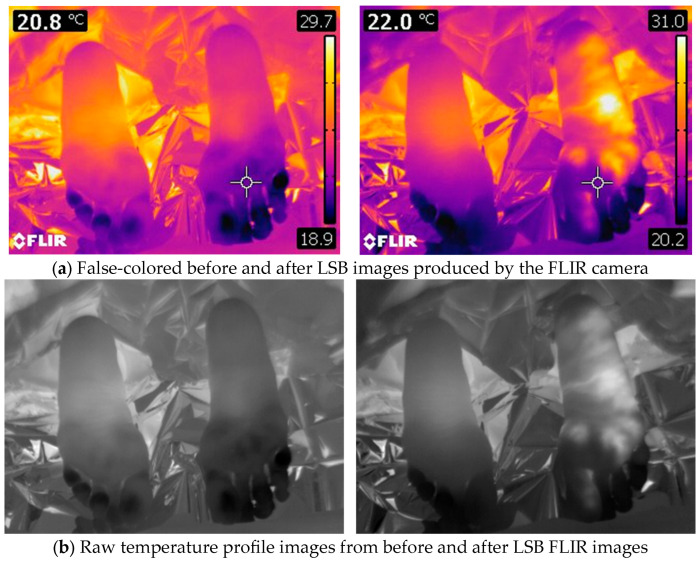
(**a**,**b**) demonstrate the processing of FLIR images using our software (proprietary software, developed using Mathematica Version 13.2. and ExifTool Version 12.54) for computing temperature differences.

**Figure 3:**
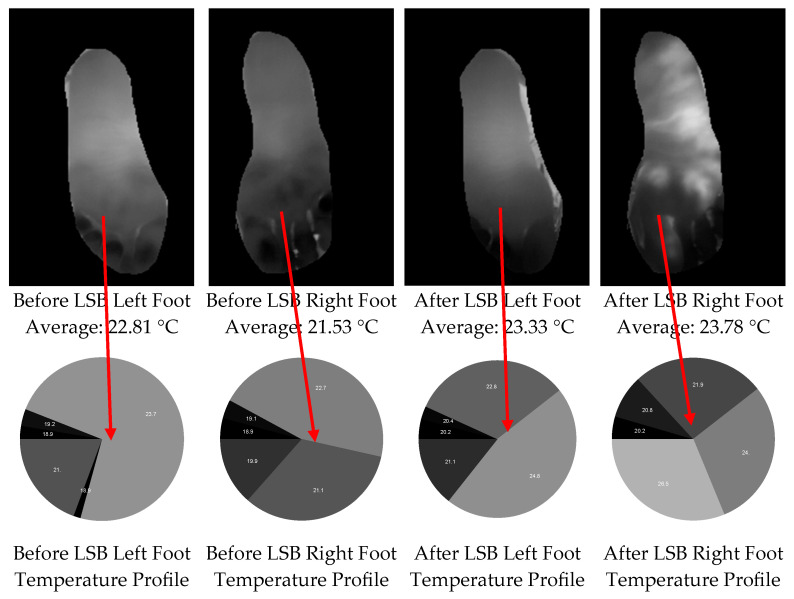
In the upper row, the created masks from the before and after FLIR images and background elimination for FLIR images are shown. In the lower row, before LSB and after LSB temperature profiles of the operated foot are shown.

**Figure 4:**
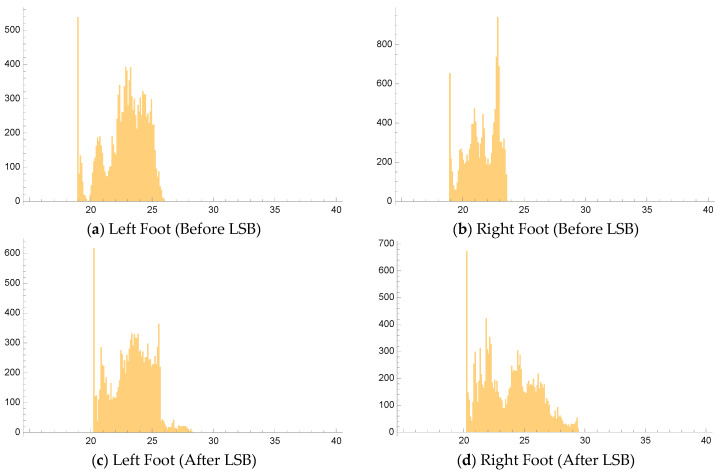
Before LSB (**a**,**b**) and after LSB (**c**,**d**) temperature histograms of operated foot with temperature bin resolution of 0.1 °C in operated foot.

We used temperature profiles obtained from infrared thermography images taken by a commercial FLIR camera for both affected and unaffected limbs (FLIR T420 camera with a resolution of 320 × 240 thermal resolution). Each picture was taken at a perpendicular angle with 1-inch space from all four sides (the positioning of the patient and the camera are shown in Figure 1). Mylar blankets were used to mitigate the influence of radiated temperature from the ankle and above. The images were saved as radiometric JPEG files, encompassing false-colored heat maps (Figure 2a) and raw thermal images (Figure 2b). The camera was normalized to record temperatures within the range of 15 °C to 40 °C. After transferring the images to a computer, we extracted the raw thermal images (Figure 2b) and implemented background removal to generate mask images for both feet (Figure 3, upper row). After isolating the two feet from the background, we extracted the temperature distribution (Figure 3, lower row) and temperature histograms (with a bin resolution of 0.1 °C, Figure 4) for both the affected and unaffected extremities. The mean temperatures for both feet were then calculated. We implemented software to process FLIR images and extract temperature profiles in Mathematica (Version Number: 13.2.0.0.). This process involved utilizing ExifTool (Version Number: 12.54), a free and open-source software program, to read the radiometric JPEG files.

We performed the LSB procedure in eligible patients diagnosed with CRPS refractory to conservative therapy. We monitored the FLIR images with a thermal camera in the lower extremities of CRPS patients throughout the LSB procedure to determine whether effective sympathetic blocks were achieved.

- In the pre-procedural period, the temperature difference between the affected and unaffected extremities is recorded.
- In the post-procedural period, the temperature difference between pre-block and post-block (5 min) time points is recorded in the affected extremity.

According to the existing literature, in a healthy population, the human body generally exhibits baseline symmetry between the extremities, making temperature differences greater than 0.5–0.7 °C between sides potentially indicative of physiological dysfunctions [20,21,22,23]. In this study, to determine warm or cold subtypes of CRPS, we followed existing literature, where a clinically significant temperature difference between extremities in CRPS patients is defined as ≥0.6 °C. If the temperature difference was <0.6 °C, we accepted it as a physiological normal variation.

In summary, based on pre-procedure temperature measurements from FLIR thermal camera images, we classified patients into three categories.

- A temperature asymmetry of ≥0.6 °C in the affected limb is classified as “warm CRPS”.
- A temperature asymmetry of ≤−0.6 °C in the affected limb is classified as “cold CRPS”.
- Any temperature difference between >−0.6 °C and <0.6 °C is considered “normal-range CRPS”.

Considering this criterion, we assigned patients to warm and cold patient groups based on the temperature differences between their affected and unaffected extremities. The temperature changes after the lumbar sympathetic blockade were then recorded for both warm and cold CRPS groups, and the results were compared. Our primary target outcome was that the mean temperature improvement in the cold CRPS patient group is expected to be higher compared to the warm CRPS patient group. The secondary target outcome was that the mean temperature elevation following the sympathetic block in the cold CRPS subtype is at least 50% or higher than the warm CRPS subtype.

### 2.7. Statistics

For the study, the sample size was determined by convenience sampling. To mitigate selection bias, all eligible patients from our records were included in the cohort. Data entry and analysis were conducted using Microsoft Excel. The distribution of pre- and post-procedural temperature differences in both initial cases and all cases (including repeated cases) was assessed using histograms. In addition, the distribution of warm and cold CRPS in both initial cases and all cases (including repeated cases) was shown as percentages. The evolution of pre- and post-procedural temperature asymmetry with repeated LSB applications, as well as the minimum, average, and maximum differences for each patient, were shown using charts. To determine the statistical significance of the results, *p*-values were calculated using a paired, 1-tailed *t*-test. Figures were also utilized to report the variation in pre- and post-procedural temperature differences for all patients.

## 3. Results

The assessment of warm and cold CRPS patients and the quantification of improvement in these patients are based on the extracted temperature profiles from preoperative and postoperative FLIR images obtained during 90 (initial and repeated) lumbar sympathetic blocks performed on 34 patients. Among the 34 patients, 26 (76.47%) were female and 8 (23.53%) were male (Table 1). All patients had a history of either surgery or trauma.

**Figure 5:**
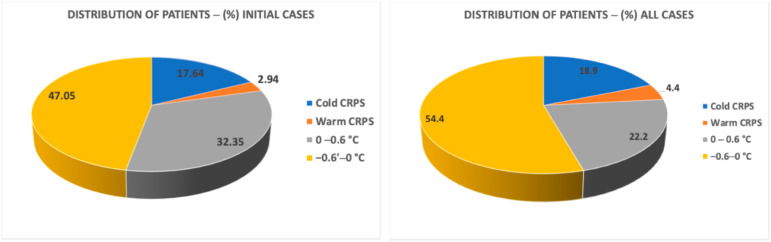
Distribution of patients by CRPS subtypes as illustrated in pie chart (left initial procedures; right all procedures).

As mentioned earlier, we classified CRPS subtypes as warm or cold based on a temperature difference ≥0.6 °C between the lower extremities at the pre-block time point. If the temperature difference was <0.6 °C, it was considered within the range of physiological normal variation. Figure 5 presents the distribution of patients classified into cold and warm CRPS subtypes based on temperature differences at the pre-block time. As shown in the figure, our results indicated that the percentages of the warm and cold CRPS groups and the normal variation group were similar in both the initial cases group and all case groups. According to our results, the percentage of cold CRPS patients was 17.64% for initial cases and 18.88% for all cases. The percentage of warm patients was 2.94% for “Initial cases” and 4.44% for “all cases”. The majority of our CRPS patients who had indications for LSB predominantly exhibited normal temperature differences in both groups (79.4% in initial cases and 76.66% in all cases).

**Figure 6:**
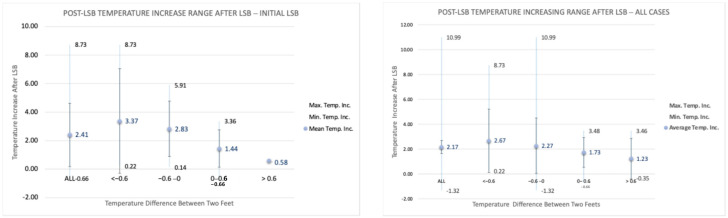
Post-LSB temperature increase levels (min, mean, max) for operated foot ((**left**) initial procedures; (**right**) all procedures) with respect to pre-procedural temperature difference between two feet.

Figure 6 presents the post-procedural temperature difference range (minimum, mean, and maximum) in the affected foot for both initial cases and all cases. The highest temperature difference that occurred was seen in one patient with 10.99 °C. Our data in all 90 cases showed that the temperature changes in the affected extremity following LSB could vary between −1.32 °C and 10.99 °C. Post-LSB temperature changes were found to be statistically significant in both the initial cases and all cases, as shown in Table 2. All *p*-values, except for the >0.6 °C group, were below 0.05, confirming statistical significance. Statistical significance could not be established for the >0.6 °C group, likely due to the small sample size (four patients). Nevertheless, this group also showed a relatively low *p*-value (~0.11), suggesting strong evidence to support our findings.

**Figure 7:**
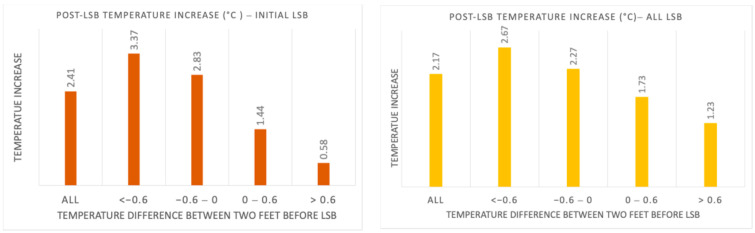
Post-LSB temperature differences (mean) for operated foot ((**left**) initial procedures; (**right**) all procedures), as illustrated in bar chart.

The analysis of the collected data showed that both primary and secondary outcomes were satisfied. The cold CRPS patients achieved higher mean temperature increases at the 5 min time point following LSB with an average of 3.37 °C and 2.67 °C for initial and all cases, respectively. The temperature increase in warm CRPS patients was lower than that of cold CRPS patients, with a mean increase of 0.58 °C and 1.23 °C for initial and all cases, respectively (Figure 7). As shown in Figure 6, the mean temperature elevation following the sympathetic block in the cold CRPS group was more than 50% higher than in the warm CRPS group, thus achieving the secondary outcome.

## 4. Discussion

The current criteria for defining a successful sympathetic block do not take into account whether a patient has cold or warm CRPS. This study compared the temperature increase following a lumbar sympathetic block between patients with cold and warm CRPS, with the expectation that the temperature increase would be greater in the cold CRPS group due to pre-existing vasoconstriction. Our findings confirmed this expectation; patients with cold CRPS experienced significantly greater temperature improvements compared to those with the warm subtype. To explore the factors contributing to the differing temperature responses to LSB in warm and cold CRPS, we will discuss the following key factors:

- Dysfunction of the sympathetic nervous system and temperature asymmetry in CRPS.
- The role of adrenoceptor activity and catecholamines in temperature asymmetry in CRPS.
- The impact of LSB and previous treatments on temperature variability in CRPS.
- The effect of LSB on temperature improvement in relation to CRPS subtypes.

### 4.1. Dysfunction of Sympathetic Nervous System and Temperature Asymmetry in CRPS

CRPS may arise from abnormal activity in the sympathetic nervous system and heightened inflammatory responses, although its exact cause remains unclear [23]. Patients with CRPS often experience fluctuations in limb temperature, alternating between warm and cold throughout the day. This temperature variation cannot be solely explained by localized inflammation, as consistent inflammation would typically result in persistent warmth [24]. Vasomotor dysfunction is a common issue among CRPS patients, with one of the most notable symptoms being temperature asymmetry [3,14]. This skin temperature imbalance may also be linked to autonomic dysfunction, where changes in circulating catecholamines can lead to limb warming or cooling [25]. In individuals with CRPS, autonomic disturbances in the affected limb can range from signs of sympathetic impairment—such as warmth and diminished vasoconstrictor reflexes—to signs of sympathetic overactivity, which may include coldness and excessive sweating [26].

### 4.2. The Role of Adrenoceptor Activity and Catecholamines in Temperature Asymmetry in CRPS

Previous studies suggest that blood flow abnormalities in affected areas can be observed due to issues with sympathetic innervation and increased sensitivity. Drummond et al. demonstrated that limbs affected by CRPS showed increased α1-adrenoceptor expression on nerve bundles in patients who experienced prolonged pain and pinprick hyperalgesia around the phenylephrine injection site compared to those with only transient pain [27]. In patients with cold CRPS, excessive vasoconstriction and reduced blood flow were observed due to increased adrenoceptor activity [25]. This heightened sensitivity of peripheral blood vessels to circulating catecholamines is likely due to an upregulation of adrenoceptors, resulting in significant vasoconstriction in the skin [28]. Conversely, the warmer skin observed in CRPS patients may be a result of suppressed sympathetic vasoconstrictor responses, leading to vasodilation [9]. This is further supported by lower norepinephrine levels detected in the affected areas [29]. Warm CRPS can be explained not only by the loss of vasoconstrictor reflexes originating in the central nervous system but also by neurogenic inflammation facilitated by peripheral mechanisms [9,10]. Vasoactive neuropeptides, which are the primary mediators of neurogenic inflammation, are responsible for vasodilation and the increased skin temperature that characterizes warm CRPS [1].

### 4.3. The Impact of the Lumbar Sympathetic Blockade and Previous Treatments on Temperature Variability in CRPS

Variability in sympathetic function, in addition to inflammation, may contribute to skin temperature asymmetry in CRPS. Studies suggest that spontaneous pain, characterized by nociceptor sensitization, is significantly influenced by the temperature at the nociceptor level [17]. Thus, the local temperature of the affected limb may be significant.

In our study, we observed a distinct distribution of subtypes at the time the patients were scheduled for LSB. Most patients treated for CRPS exhibited temperature differences considered within normal variation (less than 0.6 °C difference). Specifically, 79.4% of the initial cases and 76.66% across all cases fell within this normal range (see Figure 5). These findings suggest that conventional treatments for CRPS, administered prior to recommending minimally invasive interventions, may contribute to normalizing temperature asymmetries, as indicated by our previous research [30]. Our analysis also showed that, for both the initial cases and all cases, the percentages of patients with warm and cold CRPS subtypes were similar, as were those in the normal physiological variation group (see Figure 5). In the initial cases, 2.94% of patients presented with warm CRPS, while 17.64% had cold CRPS. In the total sample, the proportions rose to 4.44% for warm CRPS and 18.88% for cold CRPS. Therefore, we conclude that the distribution of CRPS subtypes remains relatively unchanged during the lumbar sympathetic block treatment process.

The skin temperature asymmetry observed in CRPS is a dynamic phenomenon that fluctuates in response to changes in sympathetic activity [12]. The literature indicates that physiological inhibition of cutaneous sympathetic vasoconstrictor activity can lead to an increase in skin temperature. Additionally, applying a sympathetic blockade to the affected extremity can modulate overall sympathetic outflow [17]. Following sympathetic blocks, an increase in circulation and temperature in the affected extremity is expected.

In our previous study, where the primary outcome was defined as a temperature increase of at least 1 °C in the affected limb, as detected by a FLIR camera in at least 50% of patients five minutes after the block, the results indicated that the thermal FLIR camera is a promising non-invasive tool for confirming the success of sympathetic block in the targeted limb by achieving both outcomes [19]. In our current research, we again used FLIR technology. Figure 6 presents the range of post-procedural temperature changes (minimum, mean, and maximum) in the operated foot, depending on the pre-procedural temperature difference between the two feet. The data supports our expectation of an increase in temperature following lumbar sympathetic block (LSB), with post-procedural temperature changes in the affected extremity across all 90 cases ranging from −1.32 °C to 10.99 °C. The mean temperature increase was 2.41 °C in the initial cases group, compared to 2.17 °C in the overall cases group. Furthermore, the temperature variation appears to correlate with the pre-procedural temperature difference between the two feet, as illustrated in Figure 6 and Figure 7.

### 4.4. The Effect of Lumbar Sympathetic Block on Temperature Improvement in Relation to CRPS Subtypes

The mechanisms behind temperature asymmetry differ between warm and cold CRPS patients, resulting in varying responses to lumbar sympathetic blocks and subsequent temperature changes. To better understand these differences, we analyzed temperature variations in both the warm and cold subtypes of CRPS, as well as in a normal variation group. Our primary goal was to observe a greater temperature increase in cold CRPS patients, who exhibit more pronounced vasoconstriction, compared to warm CRPS patients. As shown in Figure 7, we achieved this goal. Cold CRPS patients experienced an average temperature increase of 3.37 °C in initial cases and 2.67 °C across all cases.

In contrast, warm CRPS patients had a lower temperature increase, averaging 0.58 °C in initial cases and 1.23 °C across all cases. Additionally, the results in Figure 7 indicate that we met our secondary target outcome: the average temperature increase following sympathetic blockade in the cold CRPS subtype was 50% higher than in the warm subtype. These findings support our hypothesis that cold CRPS patients experience greater temperature improvements than warm CRPS patients after receiving a sympathetic block.

It was documented in the literature that a temperature change of 0.4 °C per minute within the first 5 min after a sympathetic block is considered an indicator of a successful block [31]. Based on this criterion, a 2 °C rise in the temperature of the affected limb is expected to signify a successful LSB at the 5-min time point following the block. However, our findings indicate that only patients with cold CRPS subtype met this success criterion, with an average temperature increase of 3.37 °C in initial cases and 2.67 °C across all cases. In contrast, patients with warm CRPS did not meet this criterion, exhibiting an average temperature increase of only 0.58 °C in initial cases and 1.23 °C across all cases. Therefore, our study suggests that temperature changes following LSB can differ between CRPS subtypes, indicating that clinicians may need to adjust their expectations for post-treatment temperature responses based on the cold or warm subtype of CRPS. Misclassifying the sympathetic block as unsuccessful, particularly in the warm subtype of CRPS, could result in the unnecessary avoidance of sympathetic blocks for therapeutic use in these patients. This concern is significant, given the current limitations in treatment options for this debilitating condition. Therefore, our findings could contribute to redefining and updating the criteria for a successful sympathetic block in warm versus cold CRPS subtypes.

### 4.5. Key Mechanisms Contributing to Greater Temperature Increases Observed in Cold CRPS Patients

Our study suggests that temperature changes after a lumbar sympathetic block may vary between different subtypes of CRPS. We aim to discuss the possible mechanisms that could explain why patients with cold CRPS typically show greater increases in temperature.

A cold extremity in chronic CRPS may arise from several factors, including increased sympathetic nervous system activity, pathological changes in the vascular wall, alterations in small nerve fibers that supply blood vessels, or endothelial dysfunction [26,32]. The transition to chronic ischemia can occur due to arterial vasospasms and capillary no-reflow, which are later consequences of endothelial damage. Impaired microcirculation leads to heightened vasoconstriction, tissue hypoxia, and metabolic acidosis in the affected limb. Reduced blood flow may stem from sympathetic dysfunction, hypersensitivity to circulating catecholamines, or endothelial dysfunction [32].

Therefore, the possible key mechanisms contributing to the greater temperature increases observed in cold CRPS patients may include the following:

- Sympathetic Vasodilation: LSB inhibits sympathetic outflow, reducing vasoconstriction, which reduces vasoconstriction. This allows for passive vasodilation, resulting in increased blood flow and elevated skin temperature.
- Restoration of Microvascular Function: Cold CRPS typically involves endothelial dysregulation and microvascular dysfunction, which can lead to ischemia in the affected limb [32,33]. By blocking the sympathetic nerves, LSB enhances capillary perfusion and mitigates the coldness caused by ischemia.
- Reduction in Pain-Induced Sympathetic Overactivity: Chronic pain and central sensitization in cold CRPS can intensify sympathetic overactivity, creating a vicious cycle of pain and vasoconstriction [26]. LSB reduces pain, leading to a secondary decrease in sympathetic tone and further enhancing blood flow.

This study has certain limitations, particularly its retrospective design, which prevented us from measuring temperature differences at the time of diagnosis before initiating conventional treatment. This study has several potential limitations in addition to its retrospective design. One significant concern is the possibility of confounding factors, such as differences in medication use and variations in symptom duration among patients. These factors may affect clinical presentations and responses to treatment. Although efforts were made to account for these variables, some residual confounding factors could still exist. To further validate these findings, future studies should be prospective, employ standardized treatment protocols, and include longer follow-up periods.

Prospective studies that measure temperature asymmetry at diagnosis and prior to the pre-LSB period would be beneficial for understanding how conventional therapies, such as physical therapy and medication management, influence the classification of patients as having cold or warm CRPS as the condition progresses. This approach would also help clarify the impact of LSB on temperature improvement according to the initial classification.

## 5. Conclusions

After administering a sympathetic block, we expect to see a more significant increase in temperature in the limb affected by cold CRPS due to vasoconstriction compared to the warm CRPS subtype. Our findings support this expectation, showing that patients with the cold CRPS subtype experienced greater temperature improvements than those with the warm subtype following the block. Currently, the criteria for determining a successful sympathetic block do not consider whether a patient has a cold or warm CRPS subtype. Therefore, our study suggests that the definition of a successful sympathetic block may need to be redefined and updated to account for cold or warm CRPS subtypes.

## Figures and Tables

**Table 1:** Demographic features of patients (# = Number of Patients).

				Duration of Pain			
	# Patients	% Patients	Average Age	3–6 Months	6–12 Months	>12 Months	Surgery/Trauma
Female	26	76.47	47.84	4	3	19	26
Male	8	23.53	39	0	1	7	8
All	34	100	43.42	4	4	26	34

**Table 2:** *p*-values for post-LSB temperature increase levels for operated foot (paired, 1-tailed *t*-test).

	ALL	<−0.6 °C	−0.6–0 °C	0–0.6 °C	>0.6 °C
*p*-values (Initial)	1.87 × 10^−7^	0.0373	1.59 × 10^−5^	0.0022	N/A
*p*-values (All)	2.68 × 10^−17^	0.0002	7.90 × 10^−10^	3.50 × 10^−7^	0.1134

## Data Availability

The datasets presented in this article are not publicly available because they are part of a clinical study and subject to privacy constrains.

## Abbreviations

The following abbreviations are used in this manuscript:

CRPS	Complex Regional Pain Syndrome
LSBs	lumbar sympathetic blocks
FLIR	forward-looking infrared
IRB	Institutional Review Board
NRS	Numerical Rating Scale
MRI	magnetic resonance imaging
CT	computerized tomography
